# Some Observations on the Anatomical Relations and Functions of the Nervous System

**Published:** 1820-04

**Authors:** 


FOR THE LONDON MEDICAL AND PHYSICAL JOURNAL.

### *Some Observations on the Anatomical Relations and Functions of the Nervous System.* By J. R.

I HAVE, as a student, taken considerable interest in the various experiments that have of late years been made on the nerves going to the viscera; but since I have studied the minute anatomy of the nerves, I have been suspicious that the experimenters have been proceeding on erroneous notions, and particularly of the anatomy of the visceral nerves.

Of this I have been more convinced on reading the letters from Dr. Philip, in your last Number, at the very time my interest in these subjects was excited by attending the lectures on the nerves by Mr. Bell.

Dr. Philip says, "the par vagum evidently conveys to the great chain of ganglions the influence of the brain:" but of this there is no proof. He then continues, "when it is divided at a short distance from its origin, the influence it conveys is cut off; but I cannot perceive," says he, "how this can at all be done by dividing the particular branch of this nerve, which goes to the cardiac portion of the stomach: before it has arrived at this place, it has formed innumerable connections with the great sympathetic and ganglions. They have received from it the influence of the brain," &c.

This shews that Dr. Wilson Philip is not aware of the points of anatomy which have been demonstrated to us,—that the grand sympathetic is not only connected to the par vagum under the jaw, but also to all the nerves at that part. He is evidently also not acquainted with the fact, that the sympathetic is connected to the brain by a succession of distinct ganglions, which are formed on the nerves proceeding from the posterior column of the spinal marrow; the same ganglions which have induced Mr. Bell, in his new system of nerves, to object in toto to the system of Bichat: for, says he, can we receive a system as correct, which takes no account of ganglia, which are in magnitude and number greatly exceeding all the other connections of the nerve called sympathetic, and merely because they are within the protection of the vertebræ. Ought not the consideration that they are guarded like parts of importance, rather prove to us that this internal range of ganglia are the more important parts of the ganglion nerves.

I do not make any remarks on the contest between Mr. Brodie and Dr. Philip, because I think it has been proved by Mr. Bell's lectures and demonstrations, that deductions from experiments on the par vagum, or any other individual nerve, must be incorrect, where the various combinations with other nerves have not been taken into account.

4

The same observations are applicable to the experiments made upon the office the nerves perform in the secretions, either of the stomach or other parts. The peculiar character of the stomach is the secretion of gastric juice, of the liver bile, and still they are both supplied by the same nerves, viz. the par vagum and the sympathetic, which are both united to the spinal marrow.

Does it not appear a very extraordinary conclusion, that, because digestion is imperfect when the par vagum is cut, that secretion depends on it. By cutting the par vagum, the function is deranged, but not more than by any other sort of violence committed on a part belonging to the organ. Would not a nail or a thorn in the stomach have the same effect? And are there not many familiar examples of the effects of injury to any part of the system on the secretions of the several viscera, as hernia, amputation, injuries of the head, &c.

On thinking of the many experiments which Dr. Philip has made, and on his proposal to make more, I cannot but regret that he has not had an opportunity of attending Mr. Bell's demonstrations of the nerves; because I am satisfied, that every one who has attended these lectures must be convinced that comparative anatomy authorises an entire new system of the nerves, very different from that on which many of the modern experimenters have been proceeding.

*Jermyn Street; March 10th.*

---

### COLLECTANEA MEDICA:

CONSISTING OF

### ANECDOTES, FACTS, EXTRACTS, ILLUSTRATIONS, &c.

*Relating to the History or the Art of Medicine, and the Auxiliary Sciences.*

Floriferis ut apes in saltibus omnia libant,
Omnia nos itidem depascimur aurea dicta.

